# Mechanisms of exercise-based rehabilitation following intracerebral hemorrhage: insights from preclinical evidence

**DOI:** 10.3389/fphar.2026.1783944

**Published:** 2026-04-21

**Authors:** Qianshi Zhang, Xu Dong, Hong Huo, Ying Zhang, Mengyun Zhang, Dongyan Wang

**Affiliations:** 1 Heilongjiang University of Chinese Medicine, Harbin, China; 2 The Second Affiliated Hospital of Heilongjiang University of Chinese Medicine, Harbin, China

**Keywords:** animal studies, exercise, intracerebral hemorrhage, literature review, mechanisms

## Abstract

**Background:**

Intracerebral hemorrhage (ICH) is associated with high mortality and disability. Although acute treatment has improved, effective interventions for long-term functional recovery remain limited. Exercise rehabilitation is recommended in clinical guidelines to improve outcomes. However, its mechanisms in ICH have not been systematically summarized. This study aimed to review the current preclinical evidence, summarize the main mechanisms of exercise rehabilitation in ICH, and provide a reference for future mechanistic research and clinical translation.

**Methods:**

We searched PubMed, EMBASE, and Web of Science from database inception to November 2025. We included studies using rodent ICH models in which exercise or rehabilitation was the main intervention and mechanistic outcomes were reported. Two reviewers independently screened the studies and extracted data. Extracted information included model type, exercise protocol, functional assessment, tissue sampling site, and mechanistic markers.

**Results:**

A total of 23 studies were included. All used collagenase-induced striatal ICH models. Exercise protocols were heterogeneous. Treadmill training was the most common intervention (10 studies). Other interventions included skilled reaching training (4 studies), acrobatic or task-based training (3 studies), swimming (2 studies), voluntary wheel running (1 study), constraint-induced movement therapy (CIMT, one study), and one study combining treadmill and voluntary exercise. One study applied treadmill preconditioning before ICH induction. Most studies reported improvement in sensorimotor function. Mechanistic findings mainly clustered into four domains: reduction of neuroinflammation (e.g., decreased IL-1β and reduced glial activation); enhanced neurotrophin-related plasticity (e.g., increased BDNF/TrkB and GAP-43, with improved dendritic and structural markers); changes related to iron/ion toxicity and oxidative injury (e.g., reduced iron load, protein oxidation, and ion imbalance); epigenetic regulation (e.g., increased histone acetylation). Task-oriented training more often reported changes in synaptic and structural plasticity, while aerobic exercise more frequently focused on inflammation and neurotrophin-related markers.

**Conclusion:**

Preclinical evidence overall supports that exercise rehabilitation may regulate multiple pathways involved in injury and repair after ICH and is associated with functional recovery. However, there is large variation in exercise type, dose, and timing across studies. Most studies are based on a single animal model. Therefore, the optimal intervention protocol remains unclear. Future studies should standardize reporting of dose and time window and strengthen the link between mechanistic markers and clinical outcomes to improve translational value.

## 1 Introduction

Stroke is a leading cause of death and disability worldwide, with over 12 million cases reported annually. Although hemorrhagic strokes account for only 10%–20% of all cases, they are catastrophic with a mortality rate as high as 50% (Zille et al., 2022). Intracerebral hemorrhage (ICH) is characterized by bleeding within the brain parenchyma, and only 25% of survivors live independently within 6 months after the ICH onset, while over 80% of survivors suffer from severe and long-term neurological disabilities (Magid-Bernstein et al., 2022). As the population ages, and with the rise of risk factors such as obesity, diabetes, and hypertension—which have been shown to correlate with an increased incidence of ICH—the number of cases is steadily increasing (Scott et al., 2022; Sun et al., 2023). The loss of social work and living abilities among ICH patients imposes a tremendous burden on individuals and the socio-economic system. In the past few decades, emergency treatment for patients with brain hemorrhage has made progress, such as blood pressure control, neurosurgery, and hemostatic strategies (Park et al., 2024), but little progress has been made in improving long-term outcomes. Thus, the management, prevention, and treatment of ICH to minimize its incidence and severity remain current challenges.

To date, exercise rehabilitation remains the most effective treatment to promote recovery after a stroke. However, our understanding of rehabilitation mainly comes from studies on ischemic stroke, with relatively less research on post-ICH exercise rehabilitation. The 2022 guidelines from the American Heart Association/American Stroke Association (AHA/ASA) suggest considering rehabilitation activities such as stretching and functional task training within 24–48 h after ICH, as rehabilitative exercise and recovery are significant determinants of ICH outcomes and quality of life (Greenberg et al., 2022). Animal and clinical evidence suggest that the degree of early improvement in rehabilitation from brain hemorrhage may be greater than that from ischemic stroke. A randomized controlled trial in China showed that initiating basic rehabilitation treatment (daily living exercises, stretching exercises, and functional training) within 48 h after brain hemorrhage can improve the survival rate and functional outcome 6 months post-stroke (Liu et al., 2014).

**FIGURE 1:**
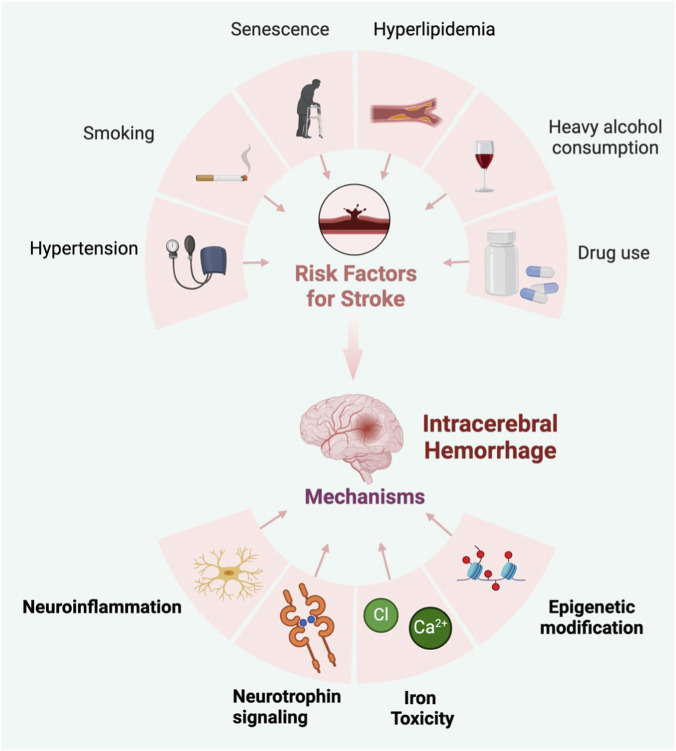
Risk factors for intracerebral hemorrhage and underlying mechanisms.

Exercise rehabilitation has been proven to improve functional outcome and reduce chronic tissue loss after ICH. To our knowledge, a comprehensive review of the potential mechanisms by which exercise can improve ICH disease activity has not been conducted. A number of animal studies have explored rehabilitation and recovery after brain hemorrhage, some of which varied the treatment parameters. Due to this limited work, it is challenging to optimize treatment protocols (such as the timing and intensity of intervention) or identify key underlying mechanisms. Therefore, we have comprehensively reviewed preclinical animal studies on exercise rehabilitation improving ICH, elucidating the potential of exercise rehabilitation as a treatment option for ICH (Figure 1). This information is crucial for understanding the fundamental science, which is essential for advancing the clinical integration of exercise rehabilitation into the routine management post-ICH.

## 2 Materials and methods

### 2.1 Information sources and search

A comprehensive literature search was performed across the following electronic databases: PubMed, EMBASE, and Web of Science, spanning from their respective inception dates up to November 2025. This search was limited to studies published in English and employed a tripartite framework of search terms encompassing: (1) populations with cerebral hemorrhage (specifically in animal models—rats and mice), (2) intervention strategies (including exercise and rehabilitation), and (3) outcome measures (focused on mechanisms). The full electronic search strategy (PubMed example) is presented in Supplementary Material (Supplementary Table 1). In addition, the reference lists of included articles were manually screened to identify potentially relevant studies not captured by database searches. Citation tracking was also performed using Web of Science.

This review was not prospectively registered, as it was designed as a narrative mechanistic synthesis.

### 2.2 Inclusion criteria

The inclusion criteria for this systematic review are defined as follows: (1) rodent model based; (2) studies where exercise, encompassing rehabilitation, is the principal intervention; (3) a control group comprising sedentary animals; and (4) investigations dedicated to the analysis of underlying mechanisms (e.g., inflammatory responses, neurotrophin signaling, iron dysregulation, oxidative stress, apoptosis, and epigenetic modifications).

### 2.3 Exclusion criteria

Studies were excluded if they involved other types of experiments, case reports, systematic reviews, or reviews. Additionally, studies with incomplete experimental data, unclear sample data, or inappropriate statistical methods were not considered. Duplicate publications or reports of identical data were also excluded.

Animal models that did not pertain to ICH, used non-rat or non-mouse models, or involved computer models were excluded. Research involving genetically modified organisms, ICH animals with complications, or featuring other treatments besides exercise was also not included. Lastly, studies that solely focused on the recovery of neurological functions without addressing broader aspects of cerebral hemorrhage were excluded.

### 2.4 Literature screening and data extraction

Firstly, two researchers (YD and (SD) independently screened the studies by reading the article titles and subsequently excluded irrelevant literature. They then read the abstracts and full texts to determine eligibility for inclusion. Data extraction included information on the authors, publication year, surgical model, exercise method, exercise stage, exercise parameters, sensorimotor assessment, site of sample collection, involved pathways, and mechanisms.

Additionally, we included information on funding sources and conflict of interest declarations in the Supplementary Material to assess potential bias risks (Supplementary Table 2).

### 2.5 Assessment of methodological quality

The risk of bias of included studies was assessed using the SYRCLE risk of bias tool. Each domain was rated as “Low”, “High”, or “Unclear”. Two reviewers independently conducted the assessment, and disagreements were resolved by consensus with a third reviewer (Hooijmans et al., 2014).

## 3 Results

### 3.1 Identification and selection of studies

**FIGURE 2:**
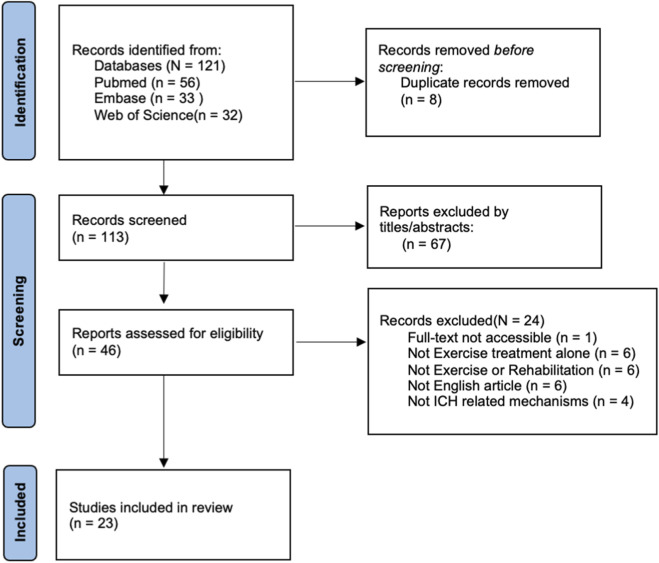
Flowchart of study selection.

A total of 121 records were identified, with 23 studies being included (Lee et al., 2003; Jin et al., 2010; Mestriner et al., 2011; Chen et al., 2012; Kim et al., 2012; Caliaperumal and Colbourne, 2014; Tamakoshi et al., 2014; Ishida et al., 2015; Takamatsu et al., 2016; Tamakoshi et al., 2016; Tamakoshi et al., 2017; Williamson et al., 2017; Zheng et al., 2019; Sato et al., 2020; Tamakoshi et al., 2020; Kinoshita et al., 2021; Tamakoshi et al., 2021; Inoue et al., 2022; Li et al., 2022; Tamakoshi et al., 2022; Maejima et al., 2023; Takamatsu et al., 2024; Tamakoshi et al., 2025). 99 studies were excluded, of which 67 were excluded based on title and abstract, and eight were excluded due to duplicate data. The remaining 24 study exclusions were insufficient access to articles, no exercise therapy alone, no exercise rehabilitation, not published in English, and no potential mechanism studies. The selection process is illustrated in Figure 2. The SYRCLE risk of bias assessment results are shown in Supplementary Material (Supplementary Figure 1).

### 3.2 Animal models of intracerebral hemorrhage

Cerebral hemorrhage demands prompt attention and urgent interventions; however, the investigation of cerebral hemorrhage within clinical environments encounters inherent limitations due to patient care priorities. In clinical research, the study of cerebral hemorrhage often necessitates the use of sophisticated imaging techniques alongside other pathological investigations. Hence, employing modeling techniques to replicate cerebral hemorrhage scenarios observed in human patients represents a judicious approach. To date, three principal methodologies have extensively facilitated cerebral hemorrhage research: autologous blood injection, collagenase-induced hemorrhage, and microsphere embolization techniques.

This review encompasses an array of studies focusing on experimentally induced Intracerebral Hemorrhage (ICH) in animal models, aiming to scrutinize the impact of motor rehabilitation interventions on functional recuperation subsequent to ICH. Notably, collagenase blood injection exclusively induced all 23 delineated ICH animal studies. The collagenase injection model, emerging as the predominant methodology in recent years, involves the administration of 0.075U–0.4U collagenase into the striatum of the animal’s basal ganglia. This procedure dissolves and disrupts small cerebral vessels, effectively simulating scenarios akin to deep cerebral hemorrhage or penetrating vascular ruptures.

### 3.3 Intervention

In the 23 included studies, treadmill exercises were chosen for in 10 instances (Lee et al., 2003; Chen et al., 2012; Takamatsu et al., 2016; Tamakoshi et al., 2020; Kinoshita et al., 2021; Tamakoshi et al., 2021; Inoue et al., 2022; Tamakoshi et al., 2022; Maejima et al., 2023; Takamatsu et al., 2024; Tamakoshi et al., 2025). Skill reaching exercises were employed in four studies (Mestriner et al., 2011; Kim et al., 2012; Caliaperumal and Colbourne, 2014; Williamson et al., 2017), with swimming training being adopted in two (Zheng et al., 2019; Li et al., 2022). Acrobatic training was the choice for three studies (Tamakoshi et al., 2014; Tamakoshi et al., 2016; Tamakoshi et al., 2017). Both Constraint-Induced Movement Therapy (CIMT) (Ishida et al., 2015) and voluntary exercises (Jin et al., 2010) were individually explored in one study each. Remarkably, one study encompassed both treadmill and voluntary exercises (Sato et al., 2020). The therapeutic modalities are summarized in Table 1. Predominantly, the studies concentrated on motor rehabilitation interventions post- ICH, with the exception of a single study that engaged in treadmill exercise as a pre-surgical intervention 6 weeks prior (Kinoshita et al., 2021).

**TABLE 1:** Structured summary of preclinical studies on exercise-based rehabilitation after intracerebral hemorrhage.

Study	Model/Animals	Exercise modality	Timing/Dose	Mechanistic focus	Main findings
Aerobic-type exercise					
Inoue et al. (2022)	Collagenase ICH; male Wistar rats	Treadmill	Post-ICH; 12 m/min, 30 min, 5 days/wk, 3 weeks	Neuroplasticity (brain, spinal cord)	Increased BDNF, GAP-43, Nogo-A, and synaptophysin
Tamakoshi et al. (2020)	Collagenase, left striatum; adult male Wistar rats	Treadmill	Early post-ICH; 9–11 m/min, 60 min, 7 days/wk, 7 days	Neuroinflammation/dendritic remodeling	Decreased IL-1β, neural cell death, and dendritic regression; increased dendritic arborization
Tamakoshi et al. (2022)	Collagenase, left striatum; adult male Wistar rats	Treadmill	Early post-ICH; 9–11 m/min, 60 min	Macrophage-related response	Decreased CD163
Kinoshita et al. (2021)	Collagenase, striatum; mice	Treadmill	Preconditioning; 10 m/min, 60 min, 7 days/wk, 5 weeks	Inflammation-related proteins/blood biomarkers	Increased CD36/Iba1, endostatin, IGFBP-2/3, MMP-9, osteopontin, and pentraxin-3
Tamakoshi et al. (2021)	Collagenase, left striatum; adult male Wistar rats	Treadmill	Early post-ICH; 9–11 m/min, 60 min	Neuroinflammation/synaptic injury	Decreased NeuN and PSD95; increased IL-1β and TGF-β1
Sato et al. (2020)	Collagenase; male Sprague–Dawley rats	Treadmill + voluntary running	Post-ICH; treadmill 10 m/min, 30 min, 4×/d; voluntary running 1224 ± 86 m/d	Activity-related plasticity/stress response	Increased FosB and corticosterone
Jin et al. (2010)	Collagenase, striatum; adult male C57BL/6 mice	Voluntary running	Post-ICH; >2000 m/d	Neurogenesis	Increased NPCs, BrdU, NeuN, and GFP
Maejima et al. (2023)	Collagenase; male Wistar rats	Treadmill	Post-ICH; 11 m/min, 30 min, 5 days/wk, 4 weeks	Epigenetic regulation	Increased histone acetylation (H3, H4)
Li et al. (2022)	Collagenase; male C57BL/6J mice	Swimming	Post-ICH; 30 min/d, 2 weeks	Metabolism/Akt-GSK3β/neuroinflammation	Decreased MI/Cr, Glu/Cr, Lac/Cr, Iba1, GFAP, Bax/Bcl-2, and mNSS; increased Cho/Cr, NAA/Cr, p-Akt/Akt, and p-GSK3β/GSK3β
Lee et al. (2003)	Collagenase; male Sprague–Dawley rats	Treadmill	Post-ICH; 2–8 m/min, 30 min, 1×/d, 10 days	Apoptosis	Decreased caspase-3
Takamatsu et al. (2016)	Collagenase; male Wistar rats	Treadmill	Post-ICH; 9 m/min, 30 min, 1×/d, 11 days	TrkB-related plasticity	Decreased dendritic regression; increased TrkB
Chen et al. (2012)	Heparinized collagenase; male C57BL/6 mice	Treadmill	Post-ICH; 40–80 m/min, 5–10 min, 14 days	BDNF/TrkB signaling	Increased BDNF and p-Y705-TrkB
Zheng et al. (2019)	Collagenase + heparin sodium; male Sprague–Dawley rats	Swimming	Post-ICH; 30 min/d, 5 days/wk, 3 weeks	NMDAR-related excitotoxicity	Decreased NR1 and NR2B
Tamakoshi et al. (2025)	Collagenase; male Wistar rats	Treadmill	Post-ICH; 11 m/min, 30 min, 1×/d, 13 days	Hippocampal plasticity	Increased BDNF
Tamakoshi et al. (2025)	Bacterial collagenase; male Wistar rats	Treadmill	Post-ICH; 11 m/min, 30 min/d, once daily, 4–27 days	Dopaminergic signaling	Increased striatal TH expression
Task-oriented training					
Tamakoshi et al. (2014)	Collagenase; adult male Wistar rats	Acrobatic tasks	Post-ICH; 4×/d, 25 days	Synaptic plasticity	Increased FosB and PSD95
Mestriner et al. (2011)	Collagenase; adult male Wistar rats	Skilled reaching	Post-ICH; 40 min/d	Astroglial response	Increased GFAP+
Ishida et al. (2015)	Collagenase (IC); adult male Wistar rats	CIMT	Post-ICH; 24 days	Neuroplasticity	Increased FosB, GAP-43, BDNF, neurites, and dendritic arborization
Caliaperumal and Colbourne. (2014)	Collagenase; male Sprague–Dawley rats	Enriched rehabilitation (skilled reaching + EE)	Post-ICH; 60 min, 5 days/wk, 7–14 days	Neuronal survival	Decreased neuronal death
Takamatsu et al. (2016)	Collagenase; male Wistar rats	Acrobatic training	Post-ICH; 4×/d, 4–28 days	AMPAR-related plasticity	Increased GluR1–4, especially GluR3 and GluR4
Kim et al. (2012)	Collagenase; male Sprague–Dawley rats	Skilled reaching	Post-ICH; 15 min, 6 days/wk, 2–4 weeks	Apoptosis/neurotrophins	Decreased Bax and caspase-3; increased BDNF, NT-3, NGF, and Bcl-2
Tamakoshi et al. (2016)	Collagenase; adult male Wistar rats	Acrobatic training	Post-ICH; 4×/d, 4–28 days	Structural plasticity	Increased MAP2 and BDNF
Williamson et al. (2017)	Collagenase; male Sprague–Dawley rats	Skilled reaching	Post-ICH; 15 min, 5 days/wk, 4 weeks	Iron/ionic deposition	Decreased fe, Cl−, calcium deposits, and protein aggregation

MMP, matrix metallopeptidase; IGFBP, Insulin-like growth factor-binding protein; MDS, motor deficit score; BWT, beam walking test; HLT, horizontal ladder tests; OFT, Open-field test; MWM, morris water maze test; mNSS, modified neurological severity score; Cr, creatine, Cho, choline, NAA, N-acetylaspartate, MI, myo-inositol, Glu, glutamate, Lac, lactate; CIMT, Constraint-induced movement therapy; IC, internal capsule; BDNF, brain-derived neurotrophic factor; GAP-43, growth-related protein 43; XFI, X-Ray Fluorescence Imaging; FTIR, fourier transform infrared imaging; ER, enriched rehabilitation; MAP2, microtubule-associated protein 2; FPT, forelimb placing test; SPRT, single pellet reaching task; NPCs, Neural progenitor cells; GFP, green fluorescent protein; PSD, postsynaptic density; Akt/GSK3β, serine-threonine kinase/glycogen synthase kinase 3β; EE, environmental enrichment; AMPAR, α-amino-3-hydroxy-5-methyl-4-isoxazolepropionic acid receptor.

### 3.4 Outcome measures

Neurofunctional evaluations across these studies were predominantly conducted using the Motor Deficit Score (MDS), Horizontal Ladder Test (HLT), Beam Walking Test (BWT), Forelimb Placing Test (FPT), and the Cylinder Test. Additional assessments reported significant reductions in experimentally induced neurofunctional deficits through the Tape Removal Task, Open-Field Test, Postural Instability Test, Forepaw Grasping, Staircase Test, and Y-Maze Test.

Significantly, only two studies indicated that motor rehabilitation did not enhance neurofunctional recovery following ICH (Tamakoshi et al., 2021; Tamakoshi et al., 2022). Conversely, the remaining investigations reported significant improvements in neurofunctional impairments post-ICH. It is hypothesized that variables such as the location of the hematoma, timing of onset, size of the hematoma, and underlying pathological conditions may influence these outcomes.

## 4 Mechanism of exercise on intracerebral hemorrhage

The pathophysiology of ICH is commonly described in terms of primary and secondary injury (Liu et al., 2025). Primary injury results from the mass effect of the hematoma and the mechanical compression of surrounding brain tissue, whereas secondary injury evolves over hours to weeks and involves a complex network of inflammatory responses, blood component toxicity, iron accumulation, oxidative stress, ionic imbalance, cell death pathways, and impaired neurovascular function. Although this framework is useful for describing disease progression, these processes are highly interconnected and cannot be fully understood as isolated events.

**FIGURE 3:**
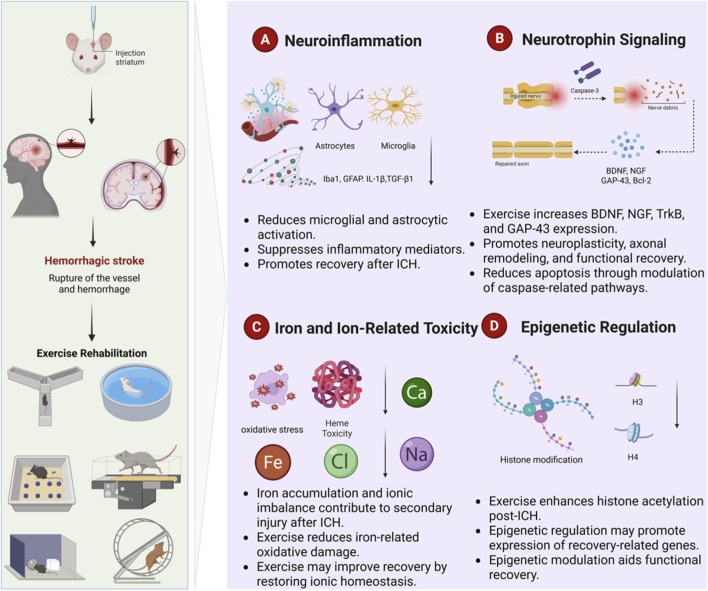
Proposed mechanistic framework of exercise after intracerebral hemorrhage. The mechanistic findings summarized in this review were organized into four interconnected domains: neuroinflammation, neurotrophin-related neural plasticity, iron and ion-related toxicity, and epigenetic regulation. These domains represent an analytical framework based on relatively consistent preclinical outcomes rather than a complete representation of the pathological network after ICH.

The present review does not attempt to reduce the pathological network of ICH to a small number of independent mechanisms. Instead, based on the relatively consistent mechanistic outcomes reported in the included preclinical studies, we organized the evidence into four analytical domains: neuroinflammation, neurotrophin-related neural plasticity, iron and ion-related toxicity, and epigenetic regulation. This classification is intended as a practical framework for synthesis rather than a complete representation of the biological processes underlying ICH. Other relevant processes, including blood-brain barrier dysfunction, hematoma clearance, vascular remodeling, and metabolic regulation, were assessed less consistently across the included studies and were therefore not incorporated as separate analytical domains in the present review (Figure 3).

### 4.1 Neuroinflammation

Neuroinflammation is a major component of secondary brain injury after intracerebral hemorrhage and has a dual role in the pathological process. On the one hand, it participates in hematoma resolution and tissue repair. On the other hand, excessive or sustained inflammatory activation can aggravate neuronal injury, promote cerebral edema, and impair functional recovery. Following ICH, blood accumulation and tissue disruption trigger a cascade of inflammatory events in the perihematomal region, including activation of microglia and astrocytes and infiltration of peripheral leukocytes, which together shape the local immune microenvironment (Xue and Yong, 2020; Tschoe et al., 2020; Alsbrook et al., 2023). Although this response contributes to clearance of blood components and cellular debris, uncontrolled neuroinflammation may amplify secondary damage. Therefore, regulation of post-ICH neuroinflammation has been considered a potential therapeutic target.

A range of pharmacological agents, including minocycline, sphingosine-1-phosphate receptor modulators, and statins, have shown neuroprotective effects in experimental ICH models, largely through suppression of detrimental inflammatory responses. However, these benefits have not been consistently translated into clinical efficacy (Xue and Yong, 2020). In contrast, accumulating evidence from experimental studies suggests that exercise rehabilitation after ICH may attenuate neuroinflammation and improve neurological recovery (Tamakoshi et al., 2020). The beneficial effects of exercise appear to be associated, at least in part, with modulation of microglial activation and inflammatory mediator profiles. In particular, exercise may promote a shift in microglial function from a predominantly pro-inflammatory state toward a more reparative phenotype, thereby reducing inflammatory injury, limiting neuronal loss, and creating a more favorable environment for recovery.

Several included studies support this view. Tamakoshi et al. (2020) compared early exercise initiated 2 days after surgery with late exercise initiated 9 days after surgery in a bacterial collagenase-induced rat model of ICH. Early exercise reduced atrophy in the sensorimotor cortex, decreased neuronal death, enhanced contralateral cortical dendritic arborization, and lowered IL-1β expression. These findings suggest that appropriately timed exercise may suppress harmful inflammatory signaling while promoting structural plasticity. Similarly, Park et al. (2010) reported that early aerobic exercise exerted neuroprotective effects and improved neurological function in rats with ICH, further supporting a link between exercise, neural activity, and post-injury repair. Evidence from swimming training studies provides additional support for an anti-inflammatory effect of exercise after ICH. Li et al. (2022) showed that a 7-day swimming intervention improved mNSS scores and exerted neuroprotective effects through activation of the serine-threonine kinase/glycogen synthase kinase 3β pathway in mice with ICH. In the same study, H-MRS demonstrated reductions in MI/Cr, Glu/Cr, and Lac/Cr ratios around the hematoma, indicating amelioration of metabolic disturbance and tissue injury. These changes were accompanied by reduced expression of Iba1 and GFAP, suggesting suppression of microglial and astrocytic activation. Together, these findings indicate that early and appropriately dosed exercise may mitigate inflammatory activation and support functional recovery after ICH.

Although the present review mainly focused on exercise interventions initiated after ICH, evidence from exercise preconditioning also supports a relationship between physical activity and inflammatory regulation. Kinoshita et al. (2021) found that 6 weeks of treadmill exercise before ICH induction promoted recovery from neurological deficits in mice. Exercise preconditioning was associated with smaller lesion volumes, increased CD36/Iba1-positive phagocytic microglia, and altered circulating levels of several survival-related factors, including MMP-9, osteopontin, endostatin, IGFBP-2, IGFBP-3, and pentraxin-3. These findings suggest that prior exercise exposure may influence the inflammatory and reparative response to hemorrhagic injury.

Blood-brain barrier dysfunction is closely linked to post-ICH neuroinflammation and represents an important component of secondary injury. Exposure to blood components, activation of inflammatory cascades, and degradation of tight junction proteins can disrupt endothelial integrity, increase BBB permeability, and contribute to perihematomal edema and inflammatory cell infiltration. In this context, preservation of BBB integrity may represent one pathway through which exercise exerts protective effects after ICH. Several exercise-associated changes identified in the present review, including reduced neuroinflammation, attenuation of apoptosis, and improved ionic homeostasis, are closely related to maintenance of neurovascular unit stability. However, BBB-related outcomes were not consistently assessed across the included studies, which limits evaluation of BBB protection as an independent mechanistic category. Current evidence therefore suggests that BBB integrity is an important component of the biological response to exercise after ICH, although its precise contribution remains to be further clarified.

In summary, neuroinflammation is a key therapeutic target after ICH, and available preclinical evidence suggests that exercise rehabilitation, particularly when initiated at an appropriate stage, may promote recovery by modulating microglial activation, reducing inflammatory injury, and stabilizing the local tissue environment. These findings support the potential value of rehabilitation strategies that are aligned with the evolving pathophysiology of ICH.

### 4.2 Neurotrophin signaling

Brain-derived neurotrophic factor (BDNF), a member of the neurotrophic factor family, is involved in the survival, differentiation, and synaptic plasticity of neurons. BDNF serves as a potential molecular basis for axonal sprouting and synaptic formation, playing a crucial role in recovery after stroke. BDNF supplementation has been shown to improve functional recovery in rat models of ICH by promoting neuroregeneration and angiogenesis, reducing tissue loss (Guan et al., 2015; Ahn et al., 2021). Additionally, the role of endogenous BDNF has been confirmed, with evidence from the cerebrospinal fluid of ICH patients and rats indicating that post-ICH BDNF treatment works by activating neurogenesis (Lin et al., 2023). Effective recovery from ICH can also be attributed to neuroplastic changes in the spinal cord, in addition to those within the brain (de Sousa Fernandes et al., 2020). BDNF is a key mediator of neuroplastic changes induced by exercise. Exercise intervention promotes motor function recovery after ICH by increasing BDNF expression in the cortex and GAP-43 expression in the spinal cord. Exercise skill training affects the expression of genes related to sensory-motor function and synaptic plasticity in the sensory-motor cortex after ICH in rats, including upregulation of mRNA expression levels of MAP2 and BDNF in the sensory-motor cortex (Tamakoshi et al., 2016).

The synthesis of BDNF is a precise mechanism for the neuroprotection of the hippocampus, hypothalamus, and cortex induced by exercise. This process plays a crucial role in glial cell generation, neurogenesis, synaptogenesis, and angiogenesis, thereby enhancing brain function. Constraint-induced movement therapy (CIMT) is a neurorehabilitation method used to improve motor function recovery after stroke (DeBow et al., 2003). Abdullahi et al. (2020) have identified the biomarker effects responsible for functional recovery after CIMT, including increased expression of BDNF, SDF-1, HIF-1α, VEGF, and GAP-43; increased number of Δ FosB-positive cells; and decreased levels of GABA and p-ERK. A rat model of ICH compared the effects of early (post-lesion day 8) and late (post-lesion days 17–24) CIMT on behavioral, histological, and molecular biological factors (Ishida et al., 2015). Early implementation of CIMT after ICH facilitated functional recovery in rats. Specifically, CIMT increased the expression of BDNF and GAP43, promoted neuronal excitation in the lesioned-side motor cortex, and led to dendritic branching of pyramidal neurons.

Proficient stretching training is a rehabilitation method that requires understanding and familiarity with the different degrees of freedom of motion devices, optimal trajectories, and inter-joint coordination. Proficient training after ICH is more effective than non-proficient training (Caliaperumal and Colbourne, 2014), possibly because this type of exercise enables the brain to adapt to new environmental conditions and solve new problems in familiar environments. Experimental evidence from a rat model of ICH suggests that proficient stretching training can increase the expression of neurotrophic factors, including BDNF, NT-3, and NGF (Kim et al., 2012). These neurotrophic factors can reduce programmed cell death and increase cell survival rates during brain injury progression. Caspase activation is a key factor in the classical pathway of cell apoptosis, with increased levels of Caspase-1 observed in mice after ICH, typically peaking at 24–72 h (Song et al., 2022). Inhibiting Caspase-1 levels can reduce BBB damage, improve neurological function, and alleviate brain edema. Following brain injury, Caspase-3 acts as the main downstream molecule in the cascade of cell apoptosis. Elevated levels of Caspase-3 induced by ICH in rats, coupled with decreased levels of Bcl-2, exacerbate damage to the cerebral hemisphere. Proficient stretching exercises lead to increased expression of Bcl-2 and decreased expression of Bax and Caspase-3 (Kim et al., 2012). BDNF reduces cell apoptosis by inhibiting the expression of Caspase-3, while excitotoxicity in the striatum activates the expression of Caspase-3 protein. Interestingly, a study involving treadmill exercise intervention in ICH rats demonstrated that treadmill exercise can inhibit the increase in lesion area caused by bleeding and the upregulation of Caspase-3 expression in the striatum (Lee et al., 2003), thereby improving recovery from central nervous system sequelae after cerebral hemorrhage. Exercise after ICH reduced the expression of caspase-3 in the contralateral hippocampus, although no direct significant differences were observed compared to the non-intervention group. However, exercise after ICH reversed the downregulation of BDNF expression in the ipsilateral hippocampus (Takamatsu et al., 2024).

BDNF-TrKB plays a crucial role in regulating synaptic function and plasticity, as well as maintaining neuronal cell survival, morphology, and differentiation. Similarly, TrkA regulates proliferation and is important for the development and maturation of the nervous system. A preclinical study validated the activation of NGF-TrkA and BDNF-TrkB signaling pathways and their involvement in brain repair processes after ICH injury. Post-ICH surgery, early treadmill exercise for 10 days enhanced the BDNF-p-Y705-TrkB signaling pathway (Chen et al., 2012). However, NGF-TrkA signaling was not modulated by treadmill rehabilitation exercise. Takamatsu et al. (2016) found that treadmill exercise can enhance motor function recovery after ICH, suppress dendritic degeneration, and upregulate TrkB expression in the ipsilateral motor cortex. The team had previously demonstrated that treadmill running exercise can improve motor function recovery in rats after ICH and alter dendritic morphology in the striatum (Takamatsu et al., 2010).

While we have provided reasonable interpretations of these results, caution must be exercised in explaining the effects of exercise on BDNF, TrkB and caspase-3 expression after ICH. Future research should assess the actual extent of basal ganglia damage through omics approaches or magnetic resonance imaging.

### 4.3 Iron toxicity

Iron plays a pivotal role in secondary brain injury following ICH. Shortly after ICH occurrence, ruptured blood vessels cause red blood cells disintegrate, releasing hemoglobin, heme, and iron into the central nervous system. Iron, one of the main products formed during hematoma degradation, induces neurotoxicity by triggering the production of free radicals and inflammatory responses, which lead to neuronal cell death in the acute phase of ICH and subsequent neurological deficits (Righy et al., 2016). The accumulation of iron initiates a cascade of neurotoxic events, marking the beginning of secondary damage post-ICH. The toxicity of iron to the central nervous system is mediated through multiple mechanisms, with redox imbalance being among the most critical. It can produce hydroxyl radicals via the Fenton reaction and promote metal-catalyzed oxidation. For example, intracranial injection of FeCl2 can result in long-term loss of neural tissue (Caliaperumal et al., 2012).

Evidence of brain injury induced by iron and heme and their protective mechanisms have led many preclinical experiments and clinical trials to focus primarily on three main objectives to reduce iron-mediated neuroinjury: clearing excess iron, inhibiting the inflammatory response caused by iron and heme, and reducing iron-mediated neuronal injury (Wan et al., 2019). In fact, iron chelation could be a potential therapeutic approach for cerebral hemorrhage. However, the efficacy of the iron chelator deferoxamine (DFX) in different animal models of ICH has shown conflicting results. Given the challenges of direct iron removal, researchers have begun exploring alternative therapeutic approaches, including exercise rehabilitation. Exercise rehabilitation has been shown to reduce lesion size, increase the complexity of neuronal dendrites, and promote the growth of astrocytic processes and density after ICH, suggesting that exercise therapy contributes to the recovery of neurons and neural networks. Moreover, exercise rehabilitation, by regulating ion balance, demonstrates its multifaceted role in neural recovery. A deeper understanding of the mechanisms behind exercise therapy for ICH could enhance treatment protocols and guide the development of new therapies.

The brain requires iron for its metabolism, and dysfunction occurs when iron homeostasis is disturbed. A preclinical study using biospectroscopy imaging investigated the mechanisms behind functional recovery after ICH facilitated by exercise (Williamson et al., 2017). Specifically, rats were induced with ICH in the striatum by injecting collagenase, with the highest total iron content at the edge of the hematoma and a significant increase in protein aggregation (oxidation). One week post-ICH, rats underwent skilled reaching training and environmental enrichment, offering multifaceted protection after cerebral hemorrhage, including reducing blood residues, decreasing hemoglobin around the hematoma and iron content, and preserving lipid and protein content in the residual striatum. The degree of protein oxidation around the hematoma was significantly reduced, indicating that exercise rehabilitation could help mitigate oxidative stress damage.

Ions and ion channels play crucial roles in the occurrence and development of ICH, being key in maintaining neural function, the formation of cerebral edema, regulating the integrity of the blood-brain barrier, and controlling neuronal death (Zhang et al., 2023). Ion imbalance, especially the disorder of chloride (Cl−), calcium (Ca2+), sodium (Na+), and potassium (K+) ions, is a critical pathophysiological process in brain injury post-ICH. Post-ICH, abnormal activity of Cl− and Na + channels may lead to the influx of these ions, causing an increase in intracellular ion concentration. This rise leads to water following the ions into the cell, causing cellular edema. The efflux of K+ might be related to neuronal hyperpolarization and cellular dysfunction. Calcium overload is directly linked to the cell death process. Post-ICH, a dysregulation of calcium homeostasis may lead to an increase in intracellular Ca2+ concentration, triggering various damaging pathways, including the activation of calpains, thus promoting apoptosis and necrosis of neural cells.

Exercise rehabilitation is an important method to promote recovery by regulating the homeostasis of ions. Specifically, for chloride ions, exercise can regulate the expression of Cl− transport proteins, helping restore the normal homeostasis of Cl−. There is evidence that exercise, by regulating chloride homeostasis, can improve functional recovery after spinal cord injury (Côté et al., 2014). This regulation helps alleviate cellular edema and restore cell volume and function.

Similarly, exercise rehabilitation may improve recovery post-ICH by regulating Cl− homeostasis (Williamson et al., 2017). Exercise rehabilitation alleviated persistent ion imbalance, particularly reducing the abnormal increase in Cl− concentration in the peri-hematoma region post-ICH without significantly affecting the imbalance of potassium ions (K+). The rehabilitative therapy also reduced the average size of calcium deposits, which are often associated with pathological cell death. Therefore, ion balance is crucial for recovery after cerebral hemorrhage, and exercise rehabilitation may help improve functional recovery by regulating mechanisms related to restoring ion balance in brain tissue. These findings underscore the potential for developing therapeutic approaches targeting ion channels and suggest that combining pharmacological treatments with exercise rehabilitation could provide new avenues for post-ICH therapy.

### 4.4 Epigenetic modification

In recent years, epigenetic mechanisms have emerged as pivotal in the field of neuroscience, particularly in the context of neural injury and repair following cerebral hemorrhage, involving the regulation of neuroplasticity and gene expression. Generally speaking, epigenetic modifications can regulate cell function and response by influencing gene expression patterns without altering the DNA sequence. This includes DNA methylation, post-translational histone modifications, non-coding RNA (ncRNA), and RNA methylation (Ma et al., 2022). Among these, common histone post-translational modifications such as acetylation, methylation, ubiquitination, and phosphorylation are crucial mechanisms for regulating chromatin structure and gene expression (Bertogliat et al., 2020). These modifications can alter the compactness of chromatin, affecting the interaction between transcription factors and DNA, thereby regulating gene expression. Following ischemic stroke and ICH, changes in histone post-translational modifications may play a role in regulating inflammation, cell apoptosis, and neural regeneration (Nishio et al., 2024).

Early research primarily focused on the decrease in histone acetylation levels following ischemic stroke and its impact on the expression of neuroprotective and reparative genes. These studies demonstrated that in the core and penumbra regions of ischemic stroke, the acetylation levels of histones H3 and H4 significantly decreased, which is closely associated with the capacity for neural injury and functional recovery (Stanzione et al., 2020). Current studies in ICH models have shown a specific reduction in the acetylation levels of histone H4. However, the use of pharmacological histone deacetylase (HDAC) inhibitors can elevate these levels (Maejima et al., 2021; Maejima et al., 2023), suggesting that modulating histone acetylation levels could offer a potential strategy for improving neural function recovery after cerebral hemorrhage (Peng et al., 2023).

Furthermore, the role of histone acetylation levels in the study of recovery mechanisms following ICH through exercise rehabilitation is increasingly recognized. Animal model studies have revealed that exercise rehabilitation can significantly increase the acetylation levels of histones H3 and H4 after ICH, supporting the potential of exercise as an effective non-pharmacological intervention in promoting neuroplasticity and functional recovery (Maejima et al., 2023). Specifically, treadmill exercises initiated 3 days post-hemorrhage and continued for approximately 4 weeks not only enhanced histone acetylation levels but also slightly improved motor function deficits. This finding underscores the significant role of exercise rehabilitation in promoting recovery after ICH through epigenetic mechanisms, even though no significant synergistic effect was observed with the combined use of exercise and the HDAC inhibitor NaB. Various factors could explain the observed differences, such as pathological background, affected cortical regions, and brain areas where samples were collected. Future research on optimizing exercise rehabilitation protocols to maximize the increase in acetylation levels, as well as further exploring the potential interactions between exercise and HDAC inhibitors, may offer more effective treatment strategies for neural recovery post-ICH.

In summary, this body of research offers a new perspective for future exploration into the epigenetic foundation of exercise rehabilitation in the recovery process following cerebral hemorrhage, particularly in terms of optimizing exercise rehabilitation programs through the modulation of epigenetic markers, thereby providing more effective treatment strategies for ICH patients. Future studies need to further investigate the interactions between exercise rehabilitation and other epigenetic mechanisms, such as DNA methylation and regulation of non-coding RNA, to deepen our understanding of the mechanisms by which exercise promotes neural recovery after cerebral hemorrhage.

### 4.5 Exercise modality and timing

The included studies showed clear heterogeneity in exercise type, training parameters, and timing of initiation. To improve comparability, we grouped the interventions into two broad categories based on training goals and movement characteristics. The first category was aerobic-type exercise, characterized by metabolic load and repetitive movement, including treadmill training, swimming, and voluntary wheel running. The second category was task-oriented training, which focused on sensorimotor integration and fine motor control, including skilled reaching, CIMT, acrobatic or balance training, and enrichment-based paradigms.

Based on reported mechanistic outcomes, aerobic-type exercise was the most common intervention. These studies mainly examined changes in inflammatory markers, neurotrophin expression, and apoptosis-related indicators, together with improvements in functional scores. For example, several treadmill and swimming studies reported reduced IL-1β levels, increased BDNF expression, and decreased caspase-3 activity. In contrast, task-oriented training was less frequently studied but more often associated with markers of synaptic remodeling and structural plasticity, such as increased FosB and PSD95 expression and greater dendritic complexity (Tamakoshi et al., 2014). These differences suggest that different exercise modalities may share common pathways but emphasize distinct biological processes, such as inflammation control, metabolic regulation, or synaptic restructuring. However, no direct head-to-head comparisons were identified. Therefore, these observations are descriptive and require further validation.

The timing of intervention also appeared to influence outcomes. Among the included studies, two that initiated forced treadmill exercise within 24 h after ICH did not show functional improvement (Tamakoshi et al., 2021). In contrast, most studies that started exercise at 48 h or later reported more consistent benefits. Taken together, this pattern suggests that exercise effects may be time-dependent.

It should be noted that exercise intensity, duration, frequency, initiation window, and outcome measures varied considerably across studies. As a result, formal comparisons between different time windows were not possible. The observations presented here are based on trend-level synthesis and highlight the need for clearer stratification of timing and standardized reporting of dose parameters in future research.

Taken together, these four domains should not be viewed as isolated mechanisms. Rather, they appear to represent interconnected components of a dynamic injury–repair network after ICH. Neuroinflammation may interact with iron dysregulation and oxidative stress to amplify secondary injury, while neurotrophin-related plasticity is more closely linked to structural and functional recovery during later stages. Epigenetic regulation may act as an upstream layer influencing the expression of genes involved in inflammation, cell survival, and synaptic remodeling. From this perspective, exercise is unlikely to act through a single pathway. Instead, its effects may depend on how it modulates the balance between injury-promoting and repair-promoting processes across different stages of ICH evolution. This stage-dependent interaction may also help explain why exercise shows beneficial effects in most studies but not when introduced too early or under inappropriate intensity.

## 5 Conclusion

This review summarizes four main mechanisms through which exercise may influence the pathological process of ICH: neuroinflammation, neurotrophin-related neural plasticity, iron-related neurotoxicity, and epigenetic regulation. Overall, current evidence supports the potential role of exercise in modulating injury and repair at multiple biological levels.

Although most studies report that exercise improves neurological function after ICH, two studies showed that exercise started within 24 h after ICH did not provide benefit and even worsened functional outcomes (Tamakoshi et al., 2021; Tamakoshi et al., 2022). In these studies, exercise was introduced during a phase when injury progression in the collagenase model was still evolving. In collagenase-induced ICH, vascular damage and hematoma expansion can continue for several hours. During this very early stage, blood–brain barrier permeability increases and inflammatory mediators rise rapidly, indicating a vulnerable tissue environment (Reyes-Esteves et al., 2025; Jia et al., 2015). Forced treadmill exercise during this window may add hemodynamic fluctuations and physiological stress, which could amplify inflammatory signaling or increase metabolic demand in peri-hematomal tissue. In contrast, studies initiating exercise at 48 h or later often reported more favorable outcomes. At this stage, inflammatory responses may begin to shift toward a reparative phenotype, and neural plasticity processes may be more active. In addition, differences in hematoma volume, collagenase dose, lesion location, exercise intensity, and duration across studies may further influence outcomes. Therefore, the conflicting findings are more likely to reflect an interaction between exercise dose and timing rather than a lack of overall therapeutic value. The effects of exercise after ICH may depend on the alignment between intervention intensity, initiation window, and the evolving stage of brain injury.

## 6 Outstanding questions

At present, randomized controlled trials of exercise or rehabilitation after ICH remain limited in number. Most clinical studies focus on early rehabilitation or early mobilization (Marek et al., 2024). These interventions are usually multi-component and individualized. They may include out-of-bed activity, bedside training, and task-oriented functional exercises. Unlike animal experiments, it is difficult in clinical settings to precisely control exercise type, intensity, duration, and start time, or to isolate single variables for comparison. In addition, clinical studies mainly evaluate functional independence, safety, and long-term outcomes. They rarely collect molecular or imaging markers that directly correspond to mechanistic pathways. As a result, clinical efficacy and biological mechanisms are often assessed at different levels.

For example, initiating rehabilitation within 48 h in hospitalized patients may improve 6-month survival and functional outcomes (Liu et al., 2014). In patients with mild to moderate ICH, early out-of-bed mobilization within 24–72 h has been shown to improve early functional independence and shorten hospital stay (Yen et al., 2020). Current guidelines also suggest considering stretching and task-oriented training within 24–48 h once the patient is medically stable. At the same time, they advise caution regarding very early or high-intensity mobilization within the first 24 h (Greenberg et al., 2022). The discrepancy between the adverse effects observed with exercise initiated within 24 h after ICH in preclinical studies and the clinical recommendation to begin rehabilitation within 24–48 h is likely related to differences in injury stage and intervention intensity. In the preclinical studies reporting no benefit or deterioration, forced treadmill exercise was initiated during the hyperacute phase of collagenase-induced ICH, when hematoma evolution, blood–brain barrier disruption, perihematomal edema, and inflammatory activation may still be ongoing. Under such conditions, early exercise may increase physiological stress and exacerbate tissue vulnerability. In contrast, clinical recommendations generally refer to graded, closely monitored, lower-intensity rehabilitation initiated after medical stabilization rather than vigorous mobilization during the first 24 h. These observations suggest that the safety and efficacy of rehabilitation after ICH depend on the interaction between intervention timing, exercise dose, and the evolving biological state of the injured brain.

In contrast, all preclinical studies included in this review were based on the collagenase-induced ICH model, and no eligible study using the autologous blood injection model was identified. Although the collagenase model is widely used and offers good reproducibility, it may be associated with more sustained bleeding, greater blood–brain barrier disruption, and a stronger inflammatory response than autologous blood injection models. By comparison, the autologous blood injection model may better reflect the effects of hematoma burden and blood-component toxicity, but is less capable of replicating progressive vascular rupture. Therefore, the mechanistic findings summarized in this review should be interpreted within the context of the collagenase-induced model. Future studies are warranted to examine whether these findings can be replicated across complementary ICH models, including autologous blood injection and, where feasible, hypertension-related spontaneous hemorrhage models.

Although understanding of how exercise modulates ICH pathology has improved in recent years, translating these mechanistic findings into safe and reproducible clinical strategies remains challenging. One key issue is that many core mechanisms are stage-dependent and can have bidirectional effects. For example, inflammation may support hematoma clearance and tissue repair, but it may also worsen secondary injury at certain time points. The net effect of exercise on these mechanisms may depend on hematoma location and size, timing of intervention, stage of injury evolution, and the patient’s baseline condition.

Current animal models still have limitations in clinical relevance. Most studies use young and healthy rodents with a single injury paradigm. These models do not fully incorporate epidemiological factors such as age, sex, and common comorbidities. They also cannot fully represent the heterogeneity of human ICH in terms of lesion location, hemorrhage volume, and disease progression. Even large animal models with gyrencephalic brains cannot fully replicate the complexity of human ICH. Moreover, blood–brain barrier integrity, hematoma clearance and phagocytosis, vascular remodeling, metabolic reprogramming, and various forms of programmed cell death also play important roles in the pathophysiology of ICH. However, these mechanisms are relatively underreported in current exercise intervention studies and have not yet formed comparable and systematic evidence.

Therefore, a bidirectional strategy may be more feasible. On the one hand, based on existing clinical evidence, it is necessary to further standardize the timing of intervention and dose characterization, and to strengthen patient stratification and safety threshold definition. On the other hand, preclinical studies should improve model design, diversify injury paradigms, and align outcome measures more closely with clinical practice, in order to reduce the gap between mechanistic research and clinical application.

## Data Availability

The original contributions presented in the study are included in the article/Supplementary Material, further inquiries can be directed to the corresponding author.
