# Supplementary material for: Mechanisms of exercise-based rehabilitation following intracerebral hemorrhage: insights from preclinical evidence
Source: Front Pharmacol. 2026 Apr 21;17:1783944. doi: 10.3389/fphar.2026.1783944 (PMC13139155; doi:10.3389/fphar.2026.1783944)

**Supplementary Materials**

**STable1: Search Strategy**

**Database: PubMed**

**Search Fields:Title/Abstract**

**Block 1: Stroke Terms**

("stroke"[Title/Abstract] OR "cerebral infarction"[Title/Abstract] OR "ischemic stroke"[Title/Abstract] OR "intracerebral hemorrhage"[Title/Abstract] OR "intracranial hemorrhage"[Title/Abstract] OR "hemorrhagic stroke"[Title/Abstract])

**Block 2: Rehabilitation & Technology Terms**

("rehabilitation"[Title/Abstract] OR "neurorehabilitation"[Title/Abstract] OR "brain-computer interface"[Title/Abstract] OR BCI[Title/Abstract] OR "rehabilitation robot*"[Title/Abstract] OR exoskeleton*[Title/Abstract] OR "virtual reality"[Title/Abstract] OR "augmented reality"[Title/Abstract] OR VR[Title/Abstract] OR AR[Title/Abstract] OR neuromodulation[Title/Abstract] OR rTMS[Title/Abstract] OR tDCS[Title/Abstract] OR "vagus nerve stimulation"[Title/Abstract] OR "functional electrical stimulation"[Title/Abstract] OR "artificial intelligence"[Title/Abstract] OR wearable*[Title/Abstract] OR "tele-rehabilitation"[Title/Abstract])

**Block 3: Animal Terms (if including animal studies)**

(animal*[Title/Abstract] OR rat*[Title/Abstract] OR mouse[Title/Abstract] OR mice[Title/Abstract] OR rodent*[Title/Abstract] OR murine[Title/Abstract] OR primate*[Title/Abstract] OR "animal model"[Title/Abstract])

**Search: (Block 1) AND (Block 2) AND (Block 3)**

**Stable2: Summary of Study Funding Sources and Potential Bias Assessment**

| **Authors** | **Funding Source** | **Type of Funding** | **Declaration of conflicting interests** | **Comments on Potential Bias** |
| --- | --- | --- | --- | --- |
| Inoue et al. (2022) | JSPS KAKENHI Grants JP17H02117 (to H.M.), JP19J23508 (to T.I.), and JP20H04048 (to H.M.) | Government | None declared | Low potential bias due to government funding. |
| Tamakoshi et al. (2020) | Grant-in-Aid for Advanced Research at Niigata University of Health and Welfare (R01B18) | Academic | None declared | Low potential bias due to academic funding. |
| Tamakoshi et al. (2022) | JSPS KAKENHI Grant Number JP 20K11269 | Government | None declared | Low potential bias due to government funding. |
| Kinoshita et al. (2021) | None | None | None declared | Low potential bias as no external funding reported. |
| Tamakoshi et al. (2021) | JSPS KAKENHI Grant Number JP 20K11269 and the Japanese Physical Therapy Association (19-A18) | Government/ Academic | None declared | Low potential bias due to government /academic funding. |
| Sato et al. (2020) | the Japan Society for the Promotion of Scientific Grant-in-Aid for Scientific Research 70757468 (C.S.) and Interdisciplinary Collaborative Research Grant for Young Scientists, Hirosaki University. | Government/ Academic | None declared | Low potential bias due to government /academic funding. |
| Jin et al. (2010) | Korea Research Foundation Grant funded by the Korean Government (MOEHRD) (KRF-2006- 331-E00006) | Government | None declared | Low potential bias due to government funding. |
| Maejima et al. (2023) | JSPS KAKENHI 18K19720 (to H.M.) and 20H04048 (to H.M.) | Government | None declared | Low potential bias due to government funding. |
| Tamakoshi et al. (2014) | Japan Society for the Promotion of Science Grant-in-Aid for Scientific Research (25750231). | Government | None declared | Low potential bias due to government funding. |
| Li et al. (2022) | Natural Science of Fujian Province (No. 2020J01759) | Government | None declared | Low potential bias due to government funding. |
| Lee, et al. (2003) | Korea Research Foundation Grant (KRF-2003-050-G00005) | Government | None declared | Low potential bias due to government funding. |
| Mestriner et al. (2011) | Brazilian Funding Agency CNPq | Government | None declared | Low potential bias due to government funding. |
| Takamatsu et al. (2016) | None | None | None declared | Low potential bias as no external funding reported. |
| Chen et al. (2012) | Shanghai Science and Technology Committee (114119b1000) and the Academy of Life Science Internal foundation for Junior Frontier Investigators (2011KIP308) | Government/ Academic | None declared | Low potential bias due to government /academic funding. |
| Ishida et al. (2015) | .Grants-in-Aid for Scientific Research from the Japan Society for the Promotion of Science (JSPS):Priority Area (C) (Nos. 23500395 and 26430020 to HH; No. 22500546 to KI); Young Scientists (B) (Nos. 26860851 to SM and 25750213 to AI); Start-up Area (No. 23700471 to AI). Japan Brain Foundation (H.H.) | Government | None declared | Low potential bias due to government funding. |
| Caliaperumal et al. (2014) | The Heart and Stroke Foundation of Canada; Senior Medical Scholar award | Government/ Academic | None declared | Low potential bias due to government /academic funding. |
| Takamatsu et al. (2017) | Japan Society for the Promotion of Science Grant-in-Aid for Scientific Research (16K16445) and a Grant-in-Aid for Advanced Research at Niigata University of Health and Welfare (H28C14). | Government/ Academic | None declared | Low potential bias due to government /academic funding. |
| Kim et al. (2012) | Kyungsung University Research Grants | Academic | None declared | Low potential bias due to academic funding. |
| Tamakoshi et al. (2016) | None | None | None declared | Low potential bias as no external funding reported. |
| Zheng et al. (2019) | None | None | None declared | Low potential bias as no external funding reported. |
| Williamson et al. (2017) | Rehabilitation Augments Hematoma Clearance and Attenuates Oxidative Injury and Ion Dyshomeostasis After Brain Hemorrhage | Government/ Academic | None declared | Low potential bias due to government /academic funding. |
| Tamakoshi et al. (2024) | Grant-in Aid for Young Scientists 18 K17739 (to YT) and Grant-in-Aid for Scientific Research (B) 20H04048 (to HM) | Government/ Academic | None declared | Low potential bias due to government /academic funding. |

Greenberg, Steven M., Wendy C. Ziai, Charlotte Cordonnier, Dar Dowlatshahi, Brandon Francis, Joshua N. Goldstein, J. Claude Hemphill, Ronda Johnson, Kiffon M. Keigher, William J. Mack, J. Mocco, Eileena J. Newton, Ilana M. Ruff, Lauren H. Sansing, Sam Schulman, Magdy H. Selim, Kevin N. Sheth, Nikola Sprigg, and Katharina S. Sunnerhagen. 2022. "2022 Guideline for the Management of Patients With Spontaneous Intracerebral Hemorrhage: A Guideline From the American Heart Association/American Stroke Association." *Stroke* 53 (7):e282-e361. doi: 10.1161/STR.0000000000000407.

Hooijmans, Carlijn R., Maroeska M. Rovers, Rob B. M. de Vries, Marlies Leenaars, Merel Ritskes-Hoitinga, and Miranda W. Langendam. 2014. "SYRCLE's risk of bias tool for animal studies." *BMC Medical Research Methodology* 14:43. doi: 10.1186/1471-2288-14-43.

Liu, Ning, Dominique A. Cadilhac, Nadine E. Andrew, Lingxia Zeng, Zongfang Li, Jin Li, Yan Li, Xuewen Yu, Baibing Mi, Zhe Li, Honghai Xu, Yangjing Chen, Juan Wang, Wanxia Yao, Kuo Li, Feng Yan, and Jue Wang. 2014. "Randomized controlled trial of early rehabilitation after intracerebral hemorrhage stroke: difference in outcomes within 6 months of stroke." *Stroke* 45 (12):3502-3507. doi: 10.1161/STROKEAHA.114.005661.

Yen, Hsiao-Ching, Jiann-Shing Jeng, Wen-Shiang Chen, Guan-Shuo Pan, Wen-Ying Chuang Pt Bs, Ya-Yun Lee, and Ting Teng. 2019. "Early Mobilization of Mild-Moderate Intracerebral Hemorrhage Patients in a Stroke Center: A Randomized Controlled Trial." *Neurorehabilitation and Neural Repair* 34 (1):72-81. doi: 10.1177/1545968319893294.

**SFigure 1: Summary of the risk of bias for the included studies**


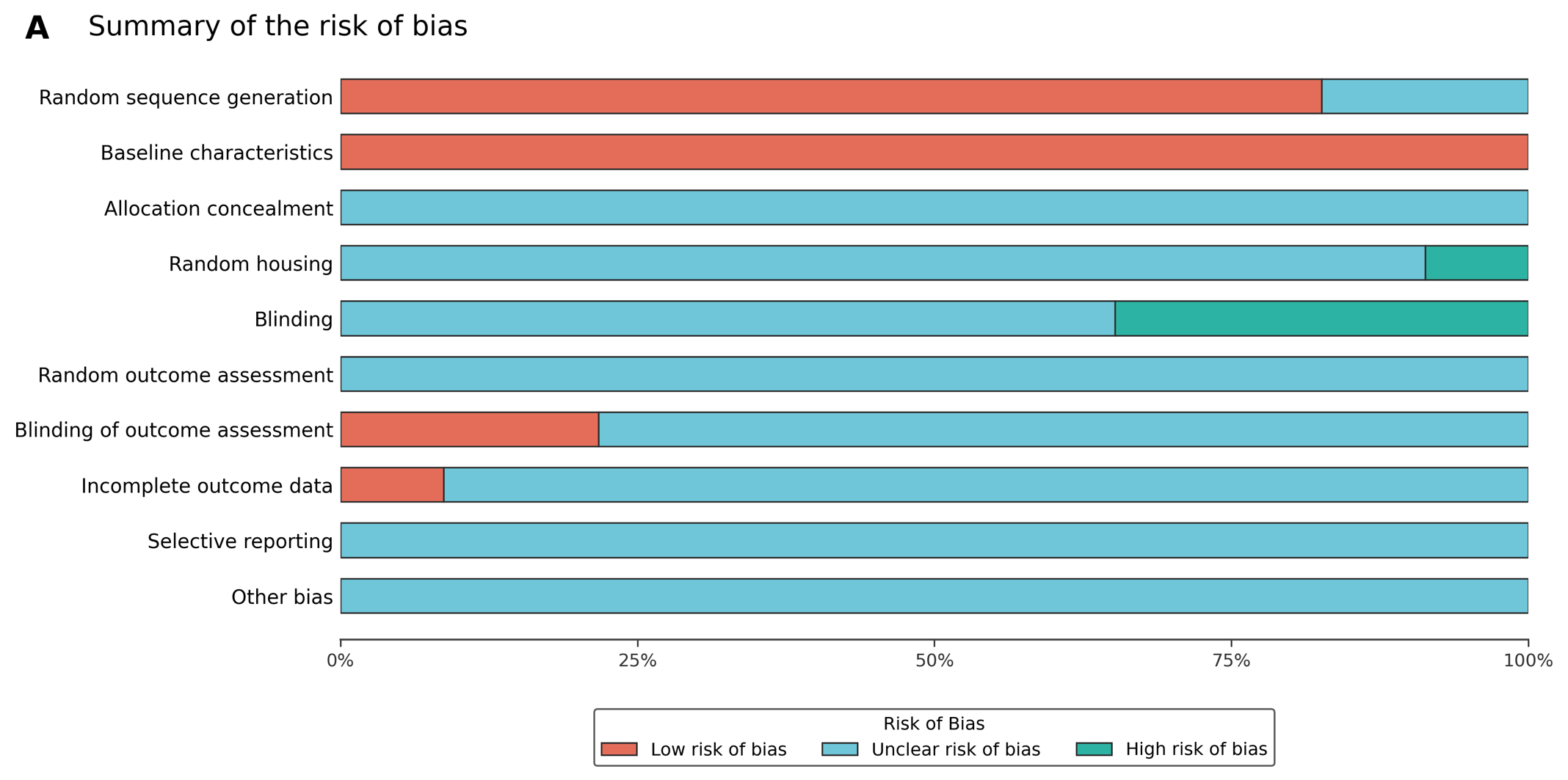

Supplement: Supplementary file 1 [file Supplementaryfile1.docx]
